# 94. Infectious Complications of Left Ventricular Assist Devices

**DOI:** 10.1093/ofid/ofab466.094

**Published:** 2021-12-04

**Authors:** Courtney Harris, Lara Coakley, Mandeep R Mehra, Hari R Mallidi, Lindsey R Baden, Ann E Woolley

**Affiliations:** Brigham and Women's Hospital, Boston, MA

## Abstract

**Background:**

Left ventricular assist devices (VAD) have significantly increased survival for patients with advanced heart failure. While advancements in devices during the past 10 years have improved thrombotic and bleeding complications, infection remains a significant cause of morbidity and mortality. We assessed the incidence and risk factors of VAD infections at our institution.

**Methods:**

A single center, retrospective study of patients who had VAD implanted between January 2007 and December 2020 was performed. Patients with concurrent right sided mechanical circulatory support devices were excluded. Patient demographics, clinical characteristics, labs, microbiology data, and antimicrobials were obtained from the electronic medical records. Clinical outcomes were adjudicated by 2 independent physicians. VAD infections were classified using the ISHLT 2011 guidelines.

**Results:**

241 patients had durable VAD implanted in this 14-year period, with a median time of 3 years follow-up. 134 (56%) patients had a clinically significant infection; 42 (31.3%) were VAD specific infections, 42 (31.3%) were VAD related, and 50 (37.4%) were non-VAD related. 95% of VAD specific infections were driveline site infections. 98% of patients with VAD related infections had a concurrent blood stream infection. Of the 50 non-VAD infections, 72% involved either a lower respiratory, urinary tract, or *Clostridium difficile* infection. Median time from VAD implantation to infection was 5 months. 44 (32.8%) had their first infection during the index hospitalization, of which 27 (61.4%) were non-VAD infections. 78 (58.2%) had one infection, compared with 38 (28.4%) who had two or more infections. 17 (12.7%) had recurrence of their initial infection and 6 (35%) occurred despite being on suppressive antibiotics. 48 of 134 (36%) infected patients were transplanted. 57 of 134 (42.5%) died compared to 33 of 107 (31%) without an infection.

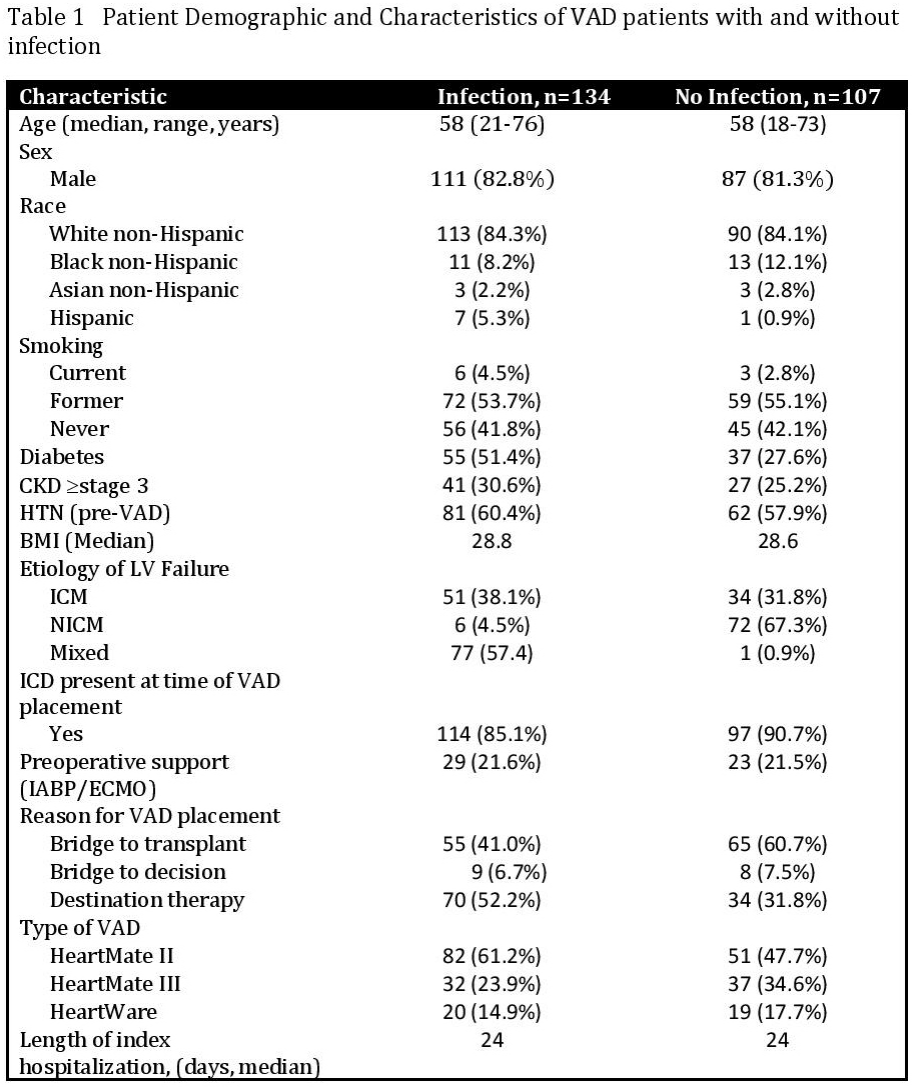

**Conclusion:**

More than half of VAD patients at our center during a 14-year time period had an infectious complication and higher mortality rate compared to those without an infectious complication. Further studies are needed to assess the immunologic risk factors for the increased risk of non-device associated infections in VAD patients.

**Disclosures:**

**Mandeep R. Mehra, MD**, **Abbott** (Consultant)**Baim Institute for Clinical Research** (Consultant)**FineHeart** (Consultant)**NupulseCV** (Consultant) **Ann E. Woolley, MD, MPH**, **COVAX** (Consultant)

